# Clinical outcomes of early salvage surgery for residual or enlarging contrast-enhancing lesions after radiotherapy in patients with glioblastoma

**DOI:** 10.3389/fonc.2026.1872660

**Published:** 2026-09-08

**Authors:** Shigeru Yamaguchi, Yukitomo Ishi, Kentaro Nishioka, Michinari Okamoto, Ryosuke Sawaya, Sogo Oki, Taiji Yamamoto, Zenichi Tanei, Shinya Tanaka, Hidefumi Aoyama, Miki Fujimura

**Affiliations:** 1 Department of Neurosurgery, Faculty of Medicine, Hokkaido University, Sapporo, Japan; 2 Department of Radiation-Oncology, Faculty of Medicine, Hokkaido University, Sapporo, Japan; 3 Department of Surgical Pathology, Hokkaido University Hospital, Sapporo, Japan; 4 Department of Cancer Pathology, Faculty of Medicine, Hokkaido University, Sapporo, Japan

**Keywords:** extent of resection, glioblastoma, overall survival, RANO resect classification, salvage surgery

## Abstract

**Background:**

Patients with glioblastoma frequently present contrast-enhancing lesions at radiotherapy completion; however, their clinical significance and optimal management remain unclear. This study evaluated the clinical outcomes of early salvage surgery performed immediately after radiotherapy completion.

**Methods:**

This retrospective single-center study included adult patients with supratentorial glioblastoma receiving standard chemoradiotherapy. The initial extent of resection was classified using postoperative magnetic resonance imaging according to the RANO *resect* framework. Early salvage surgery was defined as repeat craniotomy for safely resectable post-radiotherapy contrast-enhancing lesions before maintenance chemotherapy initiation. The Kaplan–Meier method and Cox proportional hazards models were used to analyze overall survival (OS).

**Results:**

Post-radiotherapy contrast-enhancing lesions were identified in 118 of 174 eligible patients (67.8%). Patients with lower initial RANO *resect* classes more frequently demonstrated enlarging contrast-enhancing lesions at radiotherapy completion. Early salvage surgery was performed in 17 patients (14.4%). Gross total resection (0 cm^3^ residual) and near-total resection (<1 cm^3^ residual) were achieved in 5 and 4 patients, respectively. Histopathological examination revealed viable tumor components with treatment-related changes in all resected specimens. Multivariate analysis of patients with post-radiotherapy contrast-enhancing lesions identified an independent association between early salvage surgery and longer OS (hazard ratio = 0.440, *p* = 0.021). Surgical morbidity included postoperative decline in Karnofsky Performance Status in two patients and wound-related complications in two patients.

**Conclusions:**

Early salvage surgery for post-radiotherapy contrast-enhancing lesions was independently associated with longer OS and may represent a feasible treatment option in carefully selected patients within multidisciplinary glioblastoma management.

## Introduction

The extent of tumor resection is strongly associated with prognosis in patients with glioblastoma (1, 2). The extent of resection, as assessed by postoperative magnetic resonance imaging (MRI) within three days postoperatively, is a reliable predictor of prognosis (3–6). Concurrently, postoperative neurological deterioration is an important adverse prognostic factor (7, 8). Therefore, maximum safe resection, aimed at preventing new neurological deficits, should be the goal of primary tumor resection in patients with glioblastoma (2).

Radiotherapy concomitant with temozolomide (TMZ) is the standard initial treatment for glioblastoma (9). After radiotherapy completion, maintenance TMZ therapy is administered, sometimes in combination with tumor-treating fields (10, 11). However, contrast-enhancing lesions that enlarge during or immediately post-radiotherapy are frequently encountered in clinical practice, and their biological significance often remains unclear because both tumor progression and treatment-related changes, including pseudoprogression, may present with similar imaging findings. Patients with radiological progression post-chemoradiotherapy are usually excluded from clinical trials that assess maintenance therapies (10). Therefore, a standard treatment strategy has not been established for this population. A multicenter retrospective analysis using radiotherapy-planning MRI revealed that 62.1% of patients experienced early rapid progression pre-radiotherapy (12). Further, early rapid progression was observed predominantly in patients who did not undergo gross total resection and was associated with decreasing progression-free survival (12).

Similarly, some patients show enlarging contrast-enhancing lesions during or immediately post-radiotherapy, for which the optimal management strategy remains unclear. At our institution, MRI is routinely performed immediately after radiotherapy completion in patients with glioblastoma. We consider early salvage resection through repeat craniotomy before maintenance chemotherapy initiation when residual or enlarging contrast-enhancing lesions are identified and deemed resectable. In this study, we aimed to investigate the clinical outcomes of early salvage surgery and evaluate its association with prognosis, and to discuss the feasibility of this treatment strategy.

## Materials and methods

### Patients

This retrospective study consecutively enrolled adult patients with newly diagnosed supratentorial glioblastoma treated at our institution from January 2016 to December 2025. Inclusion criteria include histopathologically confirmed glioblastoma with typical features, including microvascular proliferation and necrosis. Exclusion criteria were molecular glioblastoma or isocitrate dehydrogenase (IDH)-mutated gliomas and radiation-induced glioma. Further, the analysis excluded patients who did not receive any postoperative adjuvant therapy. This study obtained approval from our local Institutional Review Board (IRB-No: 018-0363).

### Treatment strategy

After the histopathological confirmation of glioblastoma, patients underwent radiotherapy with concomitant TMZ (9). Radiotherapy was commonly administered to the local field at a total dose of 60 Gy in 30 fractions. A hypofractionated regimen of 40 Gy in 15 fractions was administered to elderly patients (≥75 years) or those with poor performance status. Add-on bevacizumab chemotherapy was given to patients with radiologically enlarging lesions accompanied by clinical deterioration during or after radiotherapy and who were considered unresectable (13). No additional treatment was administered when pseudoprogression was strongly suspected. The final pseudoprogression diagnosis was retrospectively determined according to previously described criteria (14, 15). Maintenance TMZ was continued for up to 12 courses or until disease progression. A resectable lesion at recurrence was actively considered for surgical resection. Bevacizumab was generally administered as a last-line chemotherapy (16).

### MRI protocol

MRI was routinely performed within three days postoperatively, with most examinations obtained within 24 h. Further, an MRI was performed within 7 days after radiotherapy completion, and in most cases on the final day of radiotherapy. In our institutional practice, MRI obtained immediately after radiotherapy completion was used to assess residual or enlarging contrast-enhancing lesions before maintenance chemotherapy initiation and to enable timely consideration of salvage surgery. MRI examinations were performed using 1.5- or 3.0-T scanners and included contrast-enhanced T1-weighted imaging, T2-weighted imaging, fluid-attenuated inversion recovery, and diffusion-weighted imaging. An MRI was performed during and after maintenance therapy at 3-month intervals, in principle, or at neurological deterioration.

### Indications and timing of early salvage resection

Patients underwent additional salvage resection through repeat craniotomy before maintenance TMZ therapy when contrast-enhancing lesions were identified on MRI at radiotherapy completion and were considered safely resectable. The indication for salvage resection was determined based on MRI obtained at radiotherapy completion and subsequently multidisciplinary discussion before initiation of maintenance TMZ therapy. The indication for salvage resection was determined based on the surgical accessibility of the lesion, anticipated neurological risk, and overall clinical condition of the patient. Salvage resection was performed not only for enlarging contrast-enhancing lesions but also for non-progressive residual contrast-enhancing lesions. However, it was not performed in cases in which pseudoprogression was strongly suspected based on radiological findings and the patient’s clinical condition. Pseudoprogression was clinically suspected when radiological enlargement of contrast enhancement occurred without corresponding neurological deterioration. Conversely, enlarging contrast-enhancing lesions associated with clinical deterioration were considered less suggestive of pseudoprogression. Early salvage resection was deferred in such cases, and the final diagnosis of pseudoprogression or true progression was identified retrospectively according to follow-up MRI findings. In general, salvage resection was performed within three weeks after radiotherapy completion. Maintenance TMZ therapy was initiated within four weeks after salvage resection.

### Assessment

The primary endpoint included overall survival (OS), defined as the interval from the date of the initial operation to death or last follow-up. The extent of resection (EOR) was assessed using postoperative MRI and retrospectively reclassified according to the RANO *resect* classification (**Table 1**) (3). The RANO *resect* classification, proposed by the RANO *resect* group, categorizes the EOR based on the residual contrast-enhancing tumor volume measured on early postoperative MRI and provides a standardized framework for assessing surgical resection in glioblastoma. Volumetric analysis was conducted to calculate tumor volumes of contrast- and non-contrast-enhancing lesions, as previously described (16).

**Table 1:** Patient characteristics (N = 174).

Characteristics	No. of patients (%)
Age [median (range)]	
Median [range]	69 years [17–89 years]
Sex (Female/Male)	
Male	105 (60.3%)
Female	69 (39.7%)
Preoperative KPS	
≤ 60%	59 (33.9%)
70-80%	75 (43.1%)
90-100%	40 (23.0%)
Tumor location	
Frontal	57 (32.8%)
Parietal	36 (20.7%)
Temporal	53 (30.4%)
Occipital	6 (3.4%)
Insular	8 (4.6%)
Thalamus/Basal ganglia	14 (8.0%)
Radiotherapy	
60Gy/30Fr	126 (72.4%)
45Gy/15Fr	48 (27.6%)
Extent of resection (RANO *resect* classification)	
Class 1 (0cm^3^ CE and ≤ 5cm^3^ nCE)	6 (3.5%)
Class 2A (0cm^3^ CE and > 5cm^3^ nCE)	40 (23.0%)
Class 2B (≤ 1cm^3^ CE)	34 (19.5%)
Class 3A (≤ 5cm^3^ CE)	32 (18.4%)
Class 3B (> 5cm3 CE)	31 (17.8%)
Class 4 (No reduction of tumor volume)	31 (17.8%)

CE, contrast-enhancing tumor; KPS, Karnofsky performance status; nCE, non-contrast-enhancing tumor.

Pseudoprogression was defined as a contrast-enhancing lesion demonstrating interval enlargement that subsequently decreased in size without any additional treatment (14, 15). The final pseudoprogression diagnosis was retrospectively determined according to sequential MRI findings. The EOR of salvage resection was assessed by comparing the MRI obtained immediately before salvage surgery and within three days postoperatively.

Adverse events associated with salvage resection were assessed according to postoperative neurological deterioration and wound-related complications. Neurological deterioration is defined as a decrease in the Karnofsky Performance Status (KPS) score.

### Graphics and statistical analysis

GraphPad Prism version 8.3.0 (GraphPad Software, Inc., San Diego, CA, USA) was used to generate Kaplan–Meier survival curves. SankeyMATIC, a web-based visualization tool, was utilized to generate a Sankey diagram illustrating patient flow according to aggregated patient data.

R statistical software (version 4.2.0) was used for all statistical analyses. The log-rank test was utilized to compare survival curves. Cox proportional hazards regression models were employed to assess clinical factors associated with survival among patients with contrast-enhancing lesions identified after radiotherapy completion. Hazard ratios and 95% confidence intervals were calculated employing multivariate Cox models. A two-sided *P*-value of <0.05 indicated statistical significance.

## Results

### Patient characteristics

During the study period, 183 adult patients with supratentorial glioblastoma who met the eligibility criteria received treatment at our institution. Among them, nine were excluded because they did not receive standard concomitant chemoradiotherapy. Therefore, the final analysis included 174 patients.

**Table 1** summarizes the patient characteristics. The median age at diagnosis was 69 years (range, 17–89 years). The median follow-up period was 14.1 months (range, 1.4-95.1 months). Retrospective assessment of the EOR using the RANO *resect* classification showed the following distribution: class 1, 6 patients (3.5%); class 2A, 40 patients (23.0%); class 2B, 34 patients (19.5%); class 3A, 32 patients (18.4%); class 3B, 31 patients (17.8%); and class 4, 31 patients (17.8%).

### MRI findings after radiotherapy completion and their association with extent of resection

**Figure 1:**
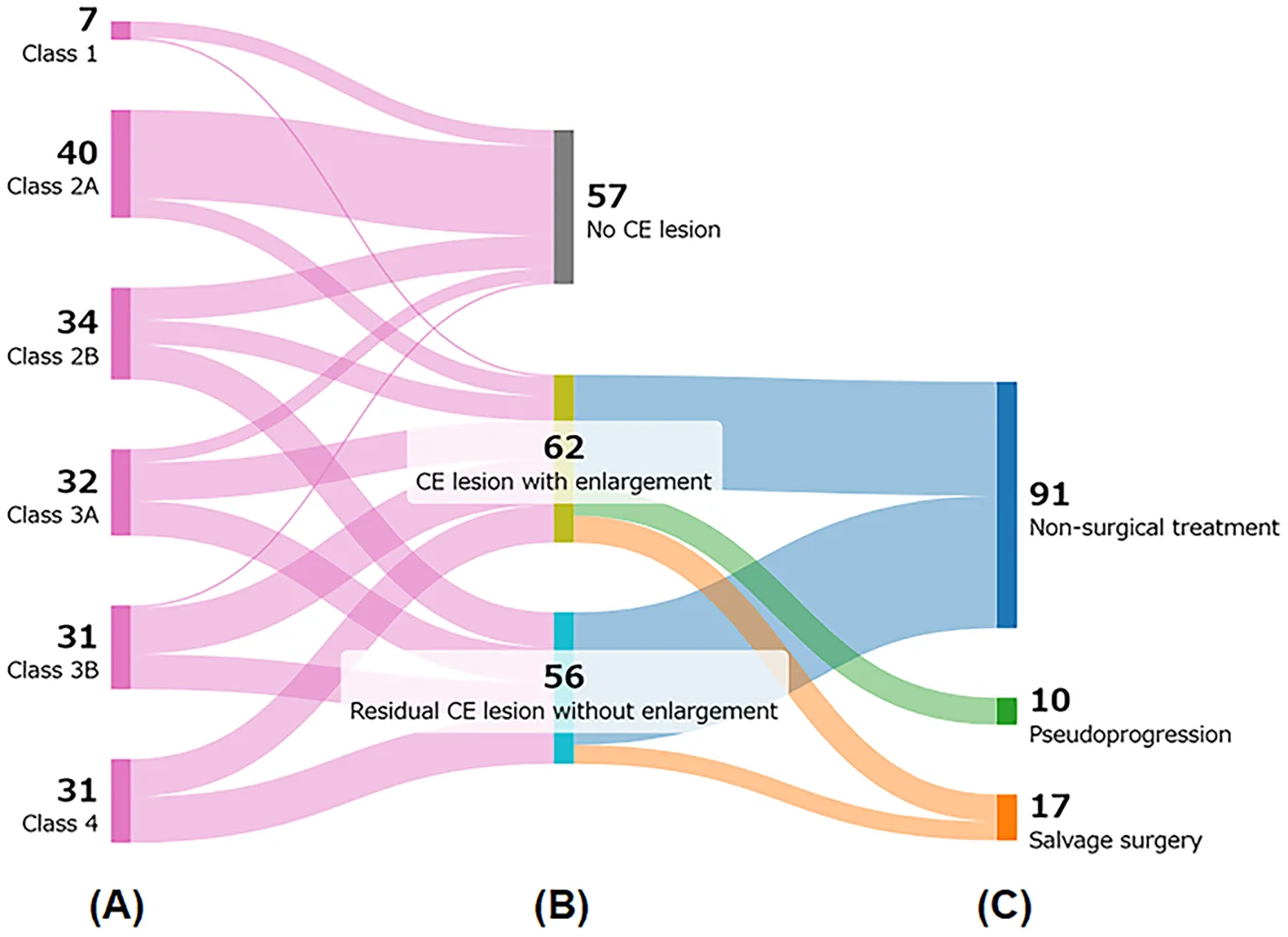
Sankey diagram illustrating patient flow according to extent of resection, MRI findings at radiotherapy completion, and subsequent management. The left column **(A)** represents the extent of resection classified according to the RANO *resect* classification. The middle column **(B)** denotes the presence and interval change of contrast-enhancing lesions at radiotherapy completion. The right column **(C)** indicates subsequent clinical interpretation and management of post-radiotherapy contrast-enhancing lesions.

**Figure 2:**
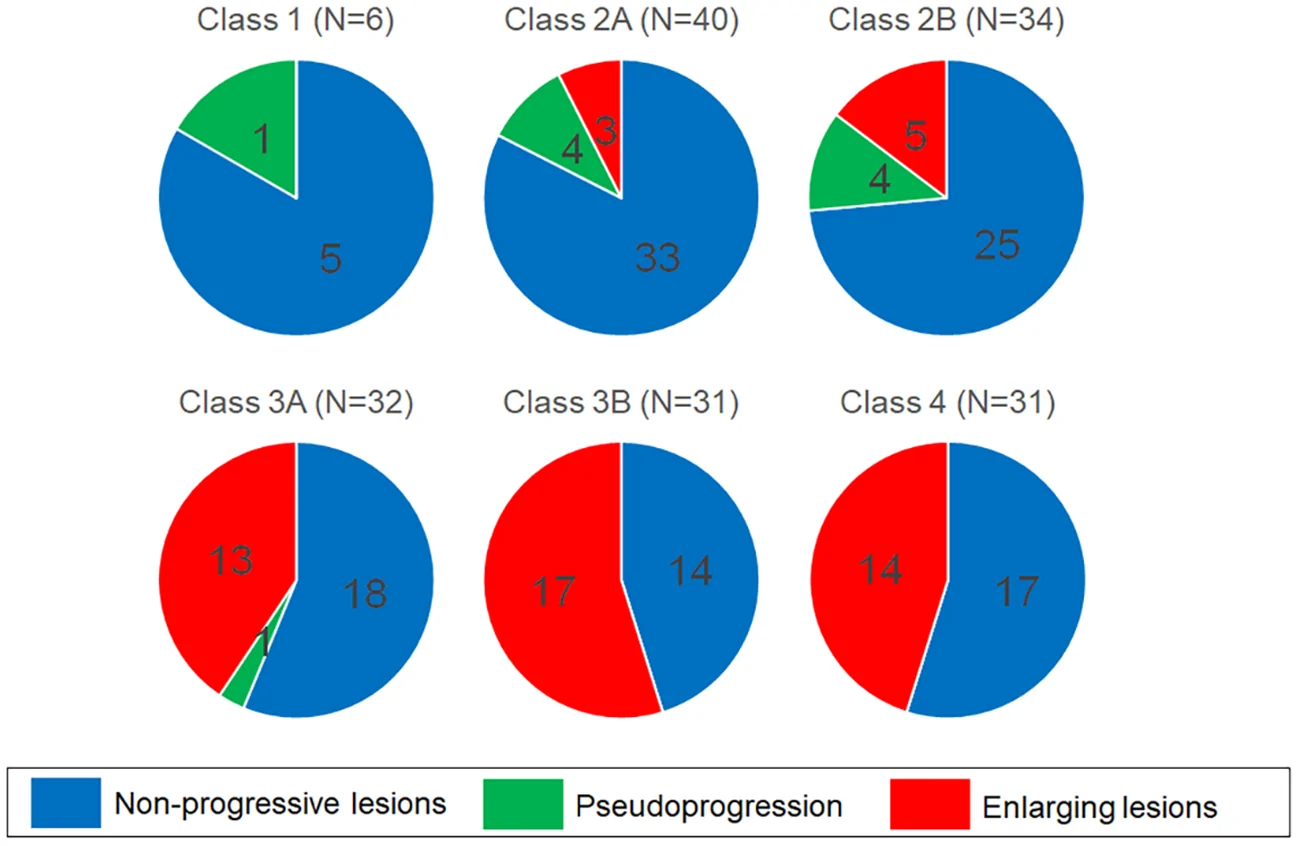
Distribution of MRI findings at radiotherapy completion according to the RANO resect classification. Pie charts demonstrate the proportion of stable or decreasing contrast-enhancing lesions (blue), pseudoprogression (green), and persistent enlarging contrast-enhancing lesions (red) within each RANO resect class.

**Figure 1** presents a Sankey diagram illustrating patient flow according to the RANO *resect* classification, MRI findings at radiotherapy completion, and subsequent clinical interpretation and management strategies. Contrast-enhancing lesions were identified on MRI post-radiotherapy in 118 patients (67.8%). Among these patients, 62 (52.5%) showed an increase in the size of contrast-enhancing lesions compared with postoperative MRI findings, whereas 56 (47.5%) demonstrated stable or decreasing contrast-enhancing lesions. The median interval between the initial surgery and radiotherapy initiation was 22 days. The median interval was 23 days and 20.5 days in patients without interval enlargement and those with enlarging lesions, respectively, with no statistically significant difference between the groups (*p* = 0.40). Among the 62 patients with enlarging contrast-enhancing lesions, 10 were ultimately diagnosed with pseudoprogression according to follow-up MRI findings. **Figure 2** presents the distribution of MRI findings at radiotherapy completion according to the RANO *resect* classification, with incidence of enlarging contrast-enhancing lesions of 17%, 18%, 26%, 45%, 55%, and 45% in classes 1, 2A, 2B, 3A, 3B, and 4, respectively. Approximately half of the patients classified as RANO *resect* classes 3–4 showed enlarging contrast-enhancing lesions at radiotherapy completion. Notably, among patients categorized as RANO *resect* classes 1–2, approximately half of the enlarging contrast-enhancing lesions (9 of 17) were ultimately diagnosed as pseudoprogression.

### Operative results of salvage surgery

Among the 118 patients with contrast-enhancing lesions identified after radiotherapy completion, 17 (14.4%) underwent additional salvage resection through repeat craniotomy. Of 17 patients, 10 had enlarging contrast-enhancing lesions, whereas 7 had residual contrast-enhancing lesions without interval enlargement. The tumors in these seven patients had initially been considered unresectable because of their location or anticipated surgical risk, but became surgically accessible after chemoradiotherapy completion. The median preoperative (post-radiotherapy) KPS score was 80% (range, 50%–90%).

#### Surgical outcomes

**Figure 3:**
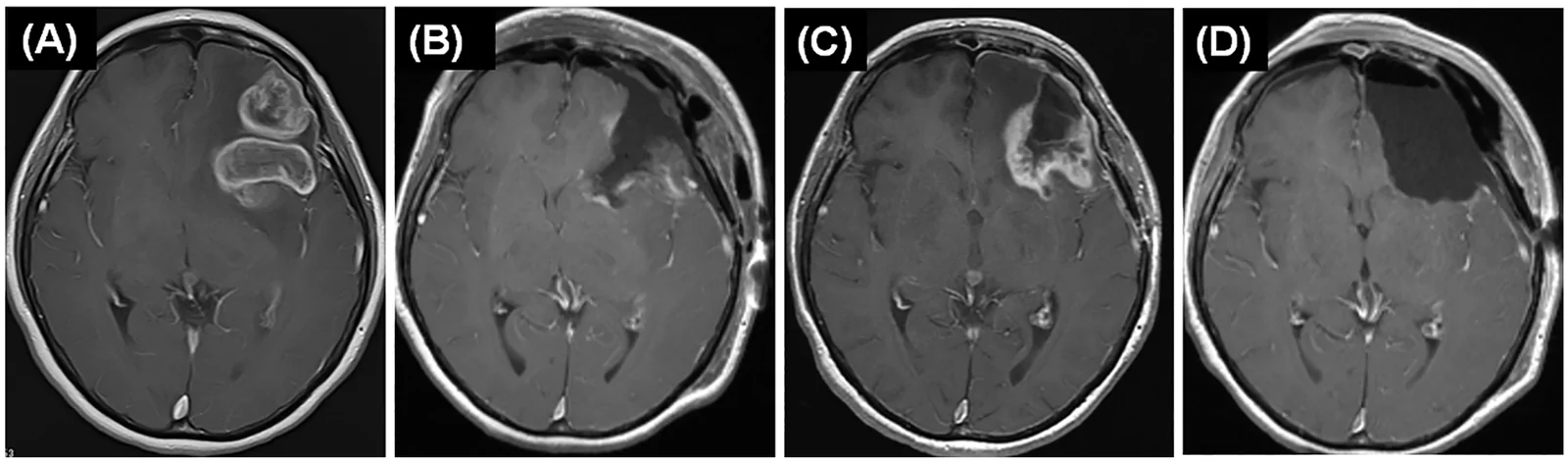
Representative case. Sequential contrast-enhanced T1-weighted magnetic resonance images of a 50-year-old female patient obtained at diagnosis **(A)**, on the day after the primary surgery **(B)**, at radiotherapy completion **(C)**, and on the day following salvage resection through repeat craniotomy **(D)**.

The mean preoperative contrast-enhancing lesion volume was 20.4 cm^3^ (range, 2.7–52.1 cm^3^), and the mean postoperative residual volume was 2.3 cm^3^ (range, 0–6.7 cm^3^). The mean EOR achieved with salvage surgery was 86.8%. Gross total resection—a residual contrast-enhancing volume of 0 cm^3^ and corresponding to RANO *resect* class 2A—was achieved in five patients (29%). Near-total resection—a residual contrast-enhancing volume of ≤1 cm^3^ and corresponding to RANO resect class 2B—was achieved in four patients (24%). **Figure 3** illustrates representative cases. Histopathological assessment demonstrated viable tumor components accompanied by varying degrees of treatment-related changes, including necrosis, gliosis, and vascular alterations, in all resected specimens.

#### Surgical complications

Postoperative neurological deterioration —a decrease in the KPS score— occurred in two patients (12%). Among these, one patient was unable to initiate maintenance TMZ therapy and subsequently transitioned to best supportive care. The remaining 16 patients successfully initiated maintenance TMZ therapy. Perioperative wound-related complications occurred in two patients, comprising wound dehiscence and bone flap infection 2 months postoperatively.

### Prognosis

**Figure 4:**
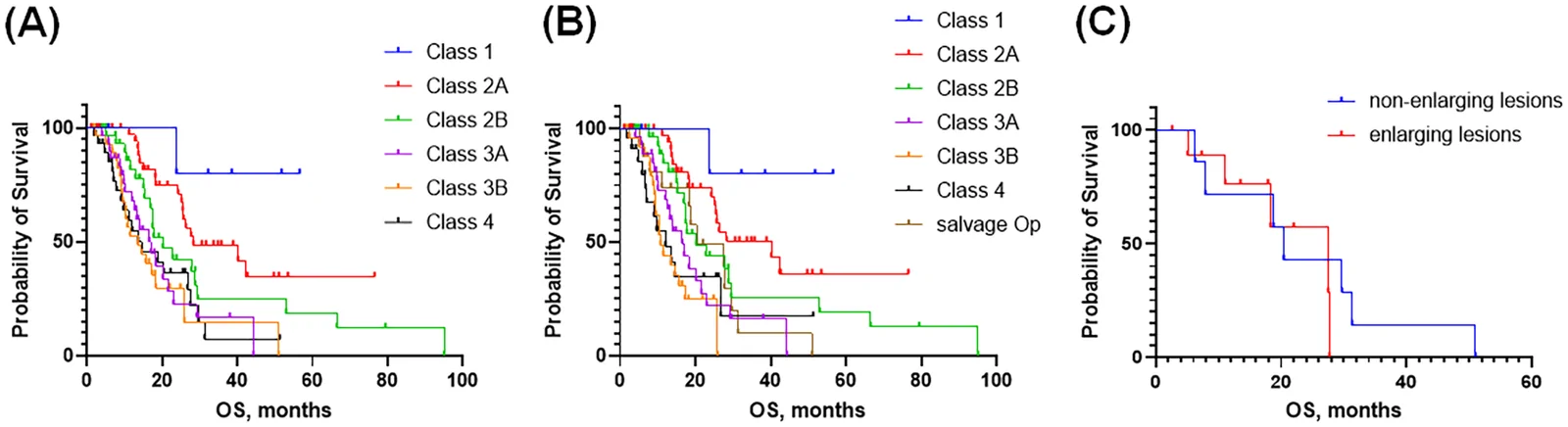
Overall survival of patients with glioblastoma. Kaplan–Meier curves demonstrating overall survival (OS) according to the RANO *resect* classification and salvage resection status. **(A)** OS of all eligible patients stratified by the RANO *resect* classification. **(B)** OS after excluding patients who underwent salvage resection from each RANO resect class, with patients who underwent salvage resection representing a separate group. The brown curve indicates the salvage resection group (“Salvage Op”). **(C)** OS of patients who underwent salvage resection stratified by the presence or absence of interval enlargement at radiotherapy completion. No statistically significant difference in OS was observed between the two groups (*p* = 0.54).

**Figure 4A** shows OS for all 174 eligible patients. The median OS by RANO *resect* class was not reached for class 1, and was 28.3 months (95% CI, 25.5 - NE (not estimable)), 20.2 months (95% CI, 16.9 - 53.0), 17.0 months (95% CI, 12.7 - 29.2), 13.5 months (95% CI, 10.3 - NE), and 14.6 months (95% CI, 9.7 - 29.7) for classes 2A, 2B, 3A, 3B, and 4, respectively. OS significantly differed across RANO *resect* classes (*p* < 0.0001). **Figure 4B** illustrates Kaplan–Meier survival curves stratified according to the RANO *resect* classification after excluding patients who underwent salvage resection. These patients were grouped into a separate “salvage resection” cohort, demonstrating a median OS of 20.4 months (95% CI, 18.3 - NE), comparable to that of patients classified as RANO *resect* class 2B (median OS, 20.2 months). In the salvage resection cohort, no significant difference in OS was observed between patients with enlarging lesions (n = 10) and those with residual non-enlarging lesions (n = 7) (*p* = 0.54) (**Figure 4C**).

Moreover, Cox proportional hazards regression analysis for OS was conducted in 108 patients with contrast-enhancing lesions identified after radiotherapy completion, excluding 10 patients who were ultimately diagnosed with pseudoprogression. Patients ultimately diagnosed with pseudoprogression were excluded because pseudoprogression represents a distinct clinical entity that may not reflect resistance to standard chemoradiotherapy. The model included the following clinical variables: (1) age, (2) RANO resect class, (3) KPS score at radiotherapy completion, (4) enlarging contrast-enhancing lesion at radiotherapy completion, and (5) salvage resection (**Table 2**). The presence of enlarging contrast-enhancing lesions at radiotherapy completion was the strongest adverse prognostic factor. The multivariate analysis identified an independent association between salvage resection and longer OS (hazard ratio: 0.424; *p* = 0.018).

**Table 2:** Univariate and multivariate analysis by Cox-proportional hazard model for overall survival.

Variable	Univariate analysis			Multivariate analysis		
	HR	95% CIs	P value	HR	95% CIs	P value
Age (> 61yo)	1.155	0.672 - 1.984	0.60	0.808	0.431 - 1.513	0.50
RANO *resect* (Class 3-4)	1.433	0.776 - 2.646	0.251	1.263	0.659 - 2.420	0.48
Post RT KPS (70-100%)	0.459	0.279 - 0.758	**0.0023**	0.491	0.276 - 0.871	**0.015**
Enlarging CE lesions	3.247	1.902 - 5.544	**<0.001**	3.631	2.041 - 6.458	**<0.001**
Salvage resection	0.637	0.337 - 1.206	0.166	0.424	0.209 - 0.862	**0.018**

CIs, confidence intervals; CE, contrast-enhancing; HR, hazard ratio; KPS, Karnofsky performance status; RT, radiotherapy. The bold values indicates a significant differences.

## Discussion

In patients with residual contrast-enhancing lesions on postoperative MRI, additional pre-radiotherapy resection has been associated with improved survival outcomes (17). However, this approach is rarely implemented in daily clinical practice. A prospective multicenter questionnaire-based study reported that no patients actually underwent additional surgery, primarily due to the perceived lack of clinical benefit, despite residual enhancing disease being considered operable in 16.3% of patients (18). In the present study, 14.4% of patients with contrast-enhancing lesions identified after radiotherapy completion underwent salvage resection. Clinicians and patients may be more inclined to consider additional surgical intervention post-radiotherapy, when disease status is clearer and further treatment decisions are required. To the best of our knowledge, this is the first clinical study to specifically evaluate early salvage resection performed immediately after radiotherapy completion in patients with glioblastoma.

Rapid early progression, defined as tumor progression occurring pre-radiotherapy, is considered a poor prognostic factor in patients with glioblastoma (19). In particular, subtotal resection is associated with a higher likelihood of rapid early progression compared with gross total resection (19). Another multicenter study revealed that progressive disease pre-radiotherapy occurred in 62.1% of patients (12). In the present study, 62 of 174 patients (35.6%) showed enlarging contrast-enhancing lesions at radiotherapy completion. Pre-radiotherapy (radiotherapy-planning) MRI was not uniformly available in our cohort; however, a substantial proportion of these cases likely represented lesion enlargement that had already begun pre-radiotherapy initiation. Furthermore, apparent enlargement of contrast enhancement immediately post-radiotherapy may partly reflect early treatment-related changes or delayed treatment response rather than true biological progression, particularly in patients with large residual tumor volumes after initial surgery. At this stage, imaging may precede the full radiological effects of chemoradiotherapy; thus, lesion enlargement interpretation at this early time point should be performed with caution. Regardless of the exact timing, both enlarging and persistent residual contrast-enhancing lesions post-radiotherapy may represent resistance to standard chemoradiotherapy. Based on this concept, we hypothesized that early additional salvage resection via repeat craniotomy might improve survival outcomes in selected patients. Consistent with this hypothesis, our results revealed that early salvage resection was independently associated with longer OS in this clinically challenging subgroup.

Pseudoprogression must be distinguished from “true” early tumor progression (14, 15). Radiological progression at four weeks after radiotherapy completion was reported in up to 42% of patients, approximately half of whom were ultimately diagnosed with pseudoprogression (20). Conversely, other studies have revealed a substantially lower pseudoprogression incidence, with only 13.3% of patients diagnosed 1 month after radiotherapy completion (15). Thus, the actual incidence of pseudoprogression in the early post-radiotherapy period remains unclear. Furthermore, the concordance between radiological assessment and subsequent histological diagnosis of pseudoprogression has been as low as 32% despite available histopathological confirmation (21), thereby emphasizing the difficulty of accurately diagnosing pseudoprogression according to imaging alone. Several clinical factors have been associated with pseudoprogression, including better neurological performance, absence of neurological deterioration, smaller baseline tumor volume, and methylated *MGMT* promoter status, in addition to radiological findings (22–25). Moreover, Melguizo–Gavilanes et al. revealed that histopathological findings in early enlarging lesions frequently showed mixed patterns, including high-grade astrocytoma and treatment-related changes, and that OS did not significantly differ according to histopathological classification (21). Altogether, these results indicate that proactive early repeated resection poses a risk of overtreatment. However, given the association between early salvage resection and longer OS observed in the present study, early additional salvage resection may represent a feasible management option in carefully selected patients, incorporating both radiological findings and overall clinical context.

Repeated craniotomy for patients with glioblastoma has been associated with a higher risk of postoperative neurological deterioration compared with primary surgery (26, 27). In particular, ischemic complications have been highlighted, with postoperative MRI showing ischemic changes in up to 80% of re-operated cases (26). Conversely, other studies have revealed no significant difference in overall complication rates between primary and repeat craniotomy, indicating that reoperation should not be uniformly discouraged when a potential benefit is anticipated (28, 29). The present study demonstrated that postoperative neurological deterioration, defined as a decrease in the KPS score, occurred in 2 of 17 patients (11.8%) who underwent early salvage resection. Of these patients, one was unable to initiate maintenance chemotherapy. These findings indicate that careful patient selection is crucial to maximize therapeutic benefit while minimizing treatment-related morbidity, although early salvage resection may offer a survival benefit.

Moreover, early repeat craniotomy performed post-radiotherapy raises concerns regarding wound-related complications. In the present study, wound-related complications occurred in 2 of 17 patients (11.8%), including wound dehiscence and bone flap infection, respectively. Repeat craniotomy in patients with glioblastoma has carried a wound-related complications risk comparable to that of primary surgery (27, 28). However, radiotherapy itself is a well-recognized risk factor for wound complications (30). Therefore, the potential increased risk of wound-related complications should be carefully considered when planning early repeat craniotomy post-radiotherapy. Furthermore, surgical-site infection is an adverse prognostic factor in patients with glioblastoma (31). Accordingly, meticulous perioperative infection prophylaxis and wound management are particularly crucial when performing early salvage resection.

This study has several limitations. First, this is a single-center retrospective analysis; thus, multicenter prospective studies are warranted to more robustly validate the observed survival association of early salvage resection. Randomized controlled trials that enroll operable patients with contrast-enhancing lesions identified at radiotherapy completion would offer the highest level of surgical evidence. However, performing such trials may be challenging because of ethical considerations. Second, molecular pathological data, particularly *MGMT* promoter methylation status, were unavailable for all patients because methylation analysis was not routinely conducted throughout the study period. *MGMT* methylation status is a well-established prognostic factor in glioblastoma and influences survival outcomes (32, 33). Because these data were unavailable for a substantial proportion of the cohort, *MGMT* status could not be incorporated into the multivariate analysis. Accordingly, the potential influence of *MGMT* methylation status on the observed survival outcomes could not be fully evaluated, which represents a limitation of this retrospective study. Previous studies have demonstrated that the EOR remains an independent prognostic factor regardless of *MGMT* status (34). Third, advanced imaging modalities, such as MR perfusion were performed only in selected patients and were not routinely incorporated into treatment decision-making throughout the study period. Therefore, treatment decisions in the present study were primarily based on conventional MRI findings and clinical status. Fourth, patient selection may have influenced the observed survival outcomes. Patients who underwent early salvage resection were selected according to surgical accessibility and overall clinical condition. Consequently, the salvage resection cohort inherently consisted of patients with operable lesions and adequate functional status, characteristics that may themselves be associated with a more favorable prognosis. Because an appropriate comparative group of patients with similarly operable lesions who did not undergo salvage resection was unavailable, selection bias cannot be excluded. In addition, only 17 patients underwent salvage resection, which limits the generalizability of present findings and may reduce the statistical robustness of subgroup analyses. Therefore, the association between salvage resection and survival should be interpreted within the context of these limitations.

## Conclusion

Contrast-enhancing lesions are frequently observed at radiotherapy completion in patients with glioblastoma and may demonstrate interval enlargement compared with postoperative MRI. Early additional salvage resection performed before maintenance chemotherapy initiation was associated with longer OS in selected patients. With careful patient selection, early salvage resection may be considered a feasible and potentially valuable surgical option in the multidisciplinary management of glioblastoma.

## Data Availability

The raw data supporting the conclusions of this article will be made available by the authors, without undue reservation.
